# *Cinnamomum cassia* Suppresses Caspase-9 through Stimulation of AKT1 in MCF-7 Cells but Not in MDA-MB-231 Cells

**DOI:** 10.1371/journal.pone.0145216

**Published:** 2015-12-23

**Authors:** Sima Kianpour Rad, M. S. Kanthimathi, Sri Nurestri Abd Malek, Guan Serm Lee, Chung Yeng Looi, Won Fen Wong

**Affiliations:** 1 Department of Molecular Medicine, Faculty of Medicine, University of Malaya, Kuala Lumpur, Malaysia; 2 University of Malaya Centre for Proteomics Research, UMCPR, Faculty of Medicine, University of Malaya, Kuala Lumpur, Malaysia; 3 Institute of Biological Sciences, Faculty of Science, University of Malaya, Kuala Lumpur, Malaysia; 4 Department of Pharmacology, Faculty of Medicine, University of Malaya, Kuala Lumpur, Malaysia; 5 Department of Medical Microbiology, Faculty of Medicine, University of Malaya, Kuala Lumpur, Malaysia; Columbia University, UNITED STATES

## Abstract

**Background:**

*Cinnamomum cassia* bark is a popular culinary spice used for flavoring and in traditional medicine. *C*. *cassia* extract (CE) induces apoptosis in many cell lines. In the present study, particular differences in the mechanism of the anti-proliferative property of *C*. *cassia* on two breast cancer cell lines, MCF-7 and MDA-MB-231, were elucidated.

**Methodology/Principal Findings:**

The hexane extract of *C*. *cassia* demonstrated high anti-proliferative activity against MCF-7 and MDA-MB-231 cells (IC_50_, 34±3.52 and 32.42 ±0.37 μg/ml, respectively). Oxidative stress due to disruption of antioxidant enzyme (SOD, GPx and CAT) activity is suggested as the probable cause for apoptosis initiation. Though the main apoptosis pathway in both cell lines was found to be through caspase-8 activation, caspase-9 was also activated in MDA-MB-231 cells but suppressed in MCF-7 cells. Gene expression studies revealed that AKT1, the caspase-9 suppressor, was up-regulated in MCF-7 cells while down-regulated in MDA-MB-231 cells. Although, AKT1 protein expression in both cell lines was down-regulated, a steady increase in MCF-7 cells was observed after a sharp decrease of suppression of AKT1. *Trans-*cinnamaldehyde and coumarin were isolated and identified and found to be mainly responsible for the observed anti-proliferative activity of CE (*Cinnamomum cassia)*.

**Conclusion:**

Activation of caspase-8 is reported for the first time to be involved as the main apoptosis pathway in breast cancer cell lines upon treatment with *C*. *cassia*. The double effects of *C*. *cassia* on AKT1 gene expression in MCF-7 cells is reported for the first time in this study.

## Introduction

Plants are generous sources of bioactive compounds in our diet. The major categories of plant-derived compounds are terpenoids, flavonoids, and alkaloids which have therapeutic effects on a variety of diseases such as cancer [1, 2]. More than 60% of currently used anticancer chemotherapeutics are derived directly or indirectly from these natural resources.

The outer bark of the evergreen *Cinnamomum cassia*, with its pungent taste and aroma, has drawn considerable attention in most parts of the world as a tasty spice. The *C*. *cassia* tree belonging to the *Lauraceae* family, originates from southern China, Bangladesh, Uganda, India, and Vietnam [2]. *C*. *cassia* is used in traditional medicine to protect against or treat many diseases as well as to maintain health and well-being. Recently, studies have indicated that *C*. *cassia* has diverse bioactivities such as antimicrobial [3], antioxidant [4] anticancer [5, 6], anti-diabetic and anti-inflammatory [7]. Both in *vitro* [8] and *in vivo* studies [9] report that *C*. *cassia* has anti-tumor activity in cervical cancer [6] colon cancer [10], myeloid cell lines [11].

The activity of antioxidant enzymes including superoxide dismutase (SOD), catalase (CAT) and glutathione peroxidase (GPx) are particularly important in cancer cell development and maintenance. In many tumor cell types CAT activity is down-regulated, whereas GPx and SOD are slightly up-regulated [12]. Plant extracts are capable of disrupting the activity of these enzymes in cancer cells to induce oxidative stress leading to death signal initiation.

Plant extracts have been shown to alter signal transduction pathways by affecting gene expression and cellular protein activity such as apoptosis [13]. Apoptosis or programmed cell death can be initiated/suppressed by activation or deactivation of several proteins such as caspase enzymes or the up/down-regulation of apoptotic genes such as AKT1, BID or p53 [14] [15]. Activation of apoptosis has been proposed as a potential mechanism for a chemotherapeutic agent to induce cancer cell death [15, 16].

In this study, sequential extraction of *C*. *cassia* bark with seven organic solvents with increasing polarity was carried out. The extracts were used to treat two breast cancer cell lines: MDA-MB-231, an estrogen negative, and MCF-7, an estrogen positive cell line. The mechanism of the observed anti-proliferative effect was further studied at the molecular level and several novel evidences are documented for the first time in this study.

## Materials and Methods

### Sample preparation

*Cinnamomum cassia* bark, obtained from the local market (NSL Distributor, Malaysia), and identified and confirmed by the Coordinator of the Botanic Garden (Rimba Ilmu), Institute of Biological Sciences, Faculty of Science, University of Malaya, was ground into a fine powder using a laboratory blender. The powder (40 g) was extracted with 200 ml of hexane. Extraction was done at 27°C ±1°C and the mixture stirred for 6 h, and extracted in triplicate. The extract was evaporated to dryness in a rotary evaporator and dissolved in DMSO.

### Cell culture

Human breast cancer cell lines, MCF-7 (estrogen-receptor positive) and MDA-MB-231 (estrogen-receptor negative) were purchased from ATCC, Va, USA. The cell lines were cultured in RPMI-1640 and DMEM (Sigma Aldrich Chemical Company, UK), respectively, and supplemented with 10% fetal bovine serum (FBS) and 1% penicillin-streptomycin solution at 37°C in a 5% CO_2_ incubator. The cells were seeded in plates at the required density per well and incubated for the desired time prior to the experiments. Cells were washed with PBS (phosphate buffered saline, pH 7.4) and incubated in fresh medium containing different concentrations of the *C*. *cassia* extract (CE). The vehicle controls received ethanol and DMSO (0.05%, v/v) in place of the extract.

### Anti-proliferative assay

The inhibition of MCF-7 and MDA-MB-231 cell proliferation on treatment with test sample was determined by the MTT (3-(4, 5-dimethylthiazol-2-yl)-2, 5-diphenyltetrazolium bromide, Sigma Aldrich Chemical Company, UK) assay [17]. Cells were seeded into a 96-well plate at a density of 5 × 10^3^ cells/well and incubated at 37°C and 5% CO_2_. After 24 h media was replaced with fresh media containing various concentrations of CE and incubated for a further 24 h.

MTT solution (10 μl of 5 mg/ml) was added and incubated for 3 h. Then the medium was removed and the remaining cells were dissolved in DMSO. The absorbance was measured at 595 nm.

### Assessment of cell viability using dual-fluorescence dyes

The viability state of the cells at IC_50_ (μg/ml) 24 h after treatment was assessed using a double fluorescent dye staining method, acridine orange (AO) and propidime iodide (PI) [18]. After the treatment period, cells were detached and washed twice using PBS to remove the remaining media. Then, 10 μl of fluorescent dyes, *viz*., AO (10 μg/ml) and PI (10 μg/ml), were added to the cellular pellet in equal volumes. Freshly stained cell suspension was placed onto a glass slide and covered with a coverslip. Slides were observed under Olympus BX50 fluorescence microscope within 30 min.

### Preparation of cell lysate

MCF-7 and MDA-MB-231 cells were seeded into 12-well plates at 5× 10^6^ cells/well and allowed to attach for 24 h. The cells were treated with 100 μg/ml of CE (IC_70_) and incubated at time points (6, 9, 12, 24 and 48 h). Total protein was extracted by sonication followed by centrifugation for 10 min at 10000 rpm at 4°C and the resulting supernatant used for the antioxidant enzyme assay. The protein concentration of the cell extract was determined by the Lowry method [19].

### Catalase (CAT) assay

The assay was performed using the Catalase Assay kit from Cayman Chemicals (USA). The assay is based on the reaction of CAT with methanol in the presence of H_2_O_2_ producing formaldehyde which is measured colorimetrically using 4-amino-3-hydrazino-5-mercapto-1, 2, 4-triazol (purpald) as the chromogen. Purpald forms a bicycle heterocycle with aldehydes, which upon oxidation changes form colorless to a purple color. CAT activity in each sample was expressed in nmol/min/ml, where one unit is defined as the amount of enzyme that caused the formation of 1.0 nmol of formaldehyde per min at 25°C.

### Superoxide dismutase (SOD) assay

This assay was performed using the superoxide dismutase Assay kit form Cayman Chemicals. This assay was performed according to the instructions provided by the manufacturer. SOD activity in each sample was expressed as U/ml, where one unit is defined as the amount of enzyme needed to exhibit 50% dismutation of the superoxide radical.

### Glutathione peroxidase (GPx) assay

This assay was performed using the GPx Assay kit from Cayman Chemicals. GPx activity was measured through a coupled reaction with glutathione reductase. This assay was performed according to the instructions provided by the manufacturer. CAT activity in each sample was expressed in nmol/min/ml, where one unit is defined as the amount of enzyme that caused the oxidation of 1.0 nmol of NADPH to NADP^+^ per minute at 25°C.

### Reactive Oxygen Species

MCF-7 and MDA-MB-231 cells were seeded into 96-well plates at a density of 5× 10^3^ cells/well and then treated with CE at the IC_50_ concentration and the ROS was measured at the time points of 6, 9, 12, 24 and 48 h. After incubation (12 h), cells were washed with PBS, and 20 μM of 2′, 7′-dichlorodihydrofluorescein diacetate (DCFH-DA) were added to each well. The absorbance was measured at 520 nm. H_2_O_2_ was used as the positive control. The data were collected from the fluorescence reader, and the average taken from the RFU (Relative Fluorescence Units) replication. Intracellular reactive oxygen species (ROS) were measured based on intracellular peroxide dependence to oxide of 2′, 7′-dichlorodihydrofluorescein diacetate (DCFH-DA) to form the fluorescent compound, 2′, 7′-dichlorofluorescein (DCF), which was measured as previously described by Halliwell (1994) [20].

### Fluorometric assay of Caspase-3/7, -8, -9 activities

MCF-7 and MDA-MB-231 cells were seeded into 96-well plates at 2.5× 10^4^cells/well. The cells were treated with CE (with or without an appropriate inhibitor) at IC_50_ and the activities were measured at the time points of 2, 8, 16, 24, and 48 h. Colchicine (1 μM) was used as the positive control for caspase-3/7 and mitomycin C was used for caspase-8 and -9.

The luminogenic substrate for caspase-3/7, caspase-8, and caspase-9 (Promega-Glo) activities were the sequences, DEVD, LETD and LEHD, respectively. Following cleavage by the appropriate caspase enzyme, the luminogenic substrate for luciferase, aminoluciferin, produces a luminescent signal. The assays were conducted according to the instructions provided by the manufacturer (Promega, US).

### Flow cytometry

Apoptosis-mediated cell death was examined by a double staining method, using a FITC-labeled Annexin V/propidium iodide (PI) apoptosis detection kit (BD Bioscience, San Jose, CA), as previously described [20]. Briefly, cells were treated for 24 h and then harvested, washed in cold phosphate-buffered saline (PBS) twice and then stained with fluoresceinisothiocyanate (FITC)-conjugated Annexin V and PI dyes. The externalization of phoshatidylserine and the permeability to PI were evaluated using a FACS Calibur flow cytometer (BD Bioscience). Data from 10,000 gated events per sample were collected. Cells in early stages of apoptosis were positively stained with Annexin V, whereas cells in late apoptosis were positively stained with both Annexin V and PI. Also, to confirm the dependence of apoptosis on caspase activity the test was done with the specific caspase inhibitor, Z-VAD-FMK.

### Apoptosis induction

For induction of apoptosis, MCF-7 and MDA-MB-231 cells were seeded into 25 cm^2^ flasks at a density of 5 x 10^6^ cells/ flask. The cells were treated with CE at IC_50_ concentrations and the activities were measured. An additional negative control can be prepared by adding the caspase inhibitor Z-VAD-FMK at 1 μl/ml to an induced culture to inhibit caspase-3 activation.

Z-VAD-FMK (carbobenzoxy-valyl-alanyl-aspartyl-[O-methyl]- fluoromethylketone) is a cell-permeant pan caspase inhibitor that irreversibly binds to the catalytic site of caspase proteases and can inhibit induction of apoptosis. For inhibition of apoptosis, Z-VAD-FMK should be added at the same time that apoptosis is induced. Z-VAD-FMK is provided at 20 mM in DMSO for convenient addition to cell culture or extracts. The assay utilizes the caspase-3 inhibitor, DEVD-FMK, conjugated to FITC (FITC-DEVD-FMK) as a marker. FITC-DEVDFMK is cell permeable, nontoxic, and irreversibly binds to activated caspase-3 in apoptotic cells. The FITC label allows for direct detection of activated caspases in apoptotic cells by fluorescence microscopy, flow cytometry, or fluorescence plate reader. The assays were conducted according to the instructions provided by the manufacturer (Biovision, USA).

### Gene expression by real-time RT-PCR

MCF-7 and MDA-MB-231cells, at a density of 5 x 10^6^ cells, were seeded into 25 cm^2^ flasks. Cells were allowed to attach overnight. Then, the cells were treated with the IC_50_ of CE and incubated for 24 h.

Total RNA extraction was conducted according to the protocol of Ultra clean tissue and cells RNA Isolation Kit (Mo-Bio, USA). The 260/280 ratio of absorbance values were measured.

The total RNA (100 ng/ml) was converted to cDNA by RT-PCR. To evaluate the expression of AKT1, p53, and Bid, gene-specific primers from TaqMan (Applied Biosystem Life technology, USA) were used. The selected genes involved in apoptosis for this study were: Hs00609632-Bid (136 bp), Hs00608023-Bcl2 (81 bp), Hs00178289-Akt1 (66 bp), and Hs01034249-p53 (108 bp). As endogenous controls, Hs01060665-β actin (63 bp) and 18 siRNA (1.9 bp) were used for biological normalizing of the selected genes. All TaqMan probes used in this study were labeled with the 6-carboxyfluorescein acronym (FAM) reporter dye at the 5’ end and a TaqMan dihydrocyclopyrroloindole tripeptide minor groove binder acronym (MGB) probe quencher at the 3’ end. The real-time polymerase chain reaction (real-time RT-PCR) was done according to the protocol of TaqMan kit (Catalog No: 4333458). After pre-heating at 95°C (5 m), the PCR was run for 40 cycles at 95°C (20 s), 59°C (40 s) and 72°C (1 m), and a final extension at 72°C (10 m). This assay was performed according to the manufacturer’s protocol.

### Western blot analysis

The cells were treated with CE (with or without caspase inhibitor) at different concentrations and also different time intervals of 0, 4, 8, 16 h at IC_50_ to measure the protein expression level of AKT1.

Western blotting was done according to reported protocol [21]. Cells were lysed in ice-cold lysis buffer containing 1% Triton X-100 and protease inhibitors. Cell lysates were clarified at 14,000 *g* for 20 minutes at 4°C. Precleared lysates were quantified using Biorad Protein Assay. 20 μg of protein were run on 10% SDS-PAGE gels and analyzed by Western blotting with the indicated antibodies followed by anti-mouse or anti-rabbit antibodies conjugated to horseradish peroxidase and enhanced chemiluminescent detection (Amersham Life Sciences, Piscataway, NJ).

Western blots were quantified using the ImageJ 1.32 software (National Institutes of Health, Bethesda, MD) after densitometric scanning of the films. In the experiment, β-Actin was chosen as the reference gene.

### Identification of active compounds

#### High performance liquid chromatography (HPLC) analysis

The experiment was performed on an Agilent 1260 infinity HPLC system consisting of a quaternary pump equipped with a 1260 autosampler (ALS), 1290 thermostat, 1260 thermostatted column compartment (TCC), 1260 diode array detector (DAD VL+), 1260 fraction collector (FC-AS) and Agilent OpenLAB CDS Chemstation for LC software.

The analytical scale analysis was carried out using a binary eluent of chromatographic grade acetonitrile (ACN) and ultrapure H_2_O under isocratic conditions: 40% ACN in H_2_O, the column used was ZORBAX Eclipse XDB-C18 (4.6 x 250 mm, 5 μm) and temperature was set to 30°C. The sample was prepared at a concentration of 5 mg/ml in methanol and filtered through a membrane filter (0.45 μm, Sartorius). The sample of 5 μl was injected into the column and peaks were detected by monitoring the UV absorbance at 254 nm. Subsequently, a higher loading of sample for preparative scale separation was attempted using the same HPLC method described above. The sample concentration was 40 mg/ml in methanol and 100 μl of sample was injected into the semi-preparative column, ZORBAX Eclipse XDB-C18 (9.4 x 250 mm, 5 μm) with a flow rate of 5 ml/min.

The selected peaks in the resultant chromatogram were collected by fraction collector and this separation procedure was conducted repeatedly. Similar fractions from each round of separation were combined and the mobile phases were evaporated in a rotary evaporator at 40°C. Then CE and the collected fractions were subjected to GC-MS analysis for identification of compounds.

#### GC-MS analysis

GCMS analysis was performed using an Agilent Technologies 6980N gas chromatograph equipped with a 5975 Mass Selective Detector (70 eV direct inlet) on fused silica capillary column, HP-5 ms (30.0 m x 0.25 mm ID x 0.25 μm film thickness). The carrier gas was helium (99.999%) at a flow rate 1 ml/min and a split ratio of 1:20. The column temperature was initially set at 60°C and was kept isothermally for 10 min, then increased by 3°C/min to 230°C and held for 1 min. The temperature of injector port and interface of mass spectrometer was programmed at 230°C and 250°C, respectively. The total ion chromatogram obtained was auto integrated by ChemStation and chemical compounds were identified by comparison with the accompanying Wiley 9^th^ edition NIST11 (W9N11) mass spectral library, USA.

### Statistical analysis

All the data were expressed as mean ± standard deviation (SD) of three replicates. Statistical analyses were performed by one-way analysis of variance (ANOVA) with Tukey’s multiple comparisons and the Student’s *t*-test. Data were analysed with SPSS (Statistical Package for the Social Sciences) 15.0 for Windows. The mean and standard deviation of means were calculated. The values of *p* < 0.05 were considered as significant.

## Results

### Effect of CE on the growth of cancer cell lines

**Fig 1:**
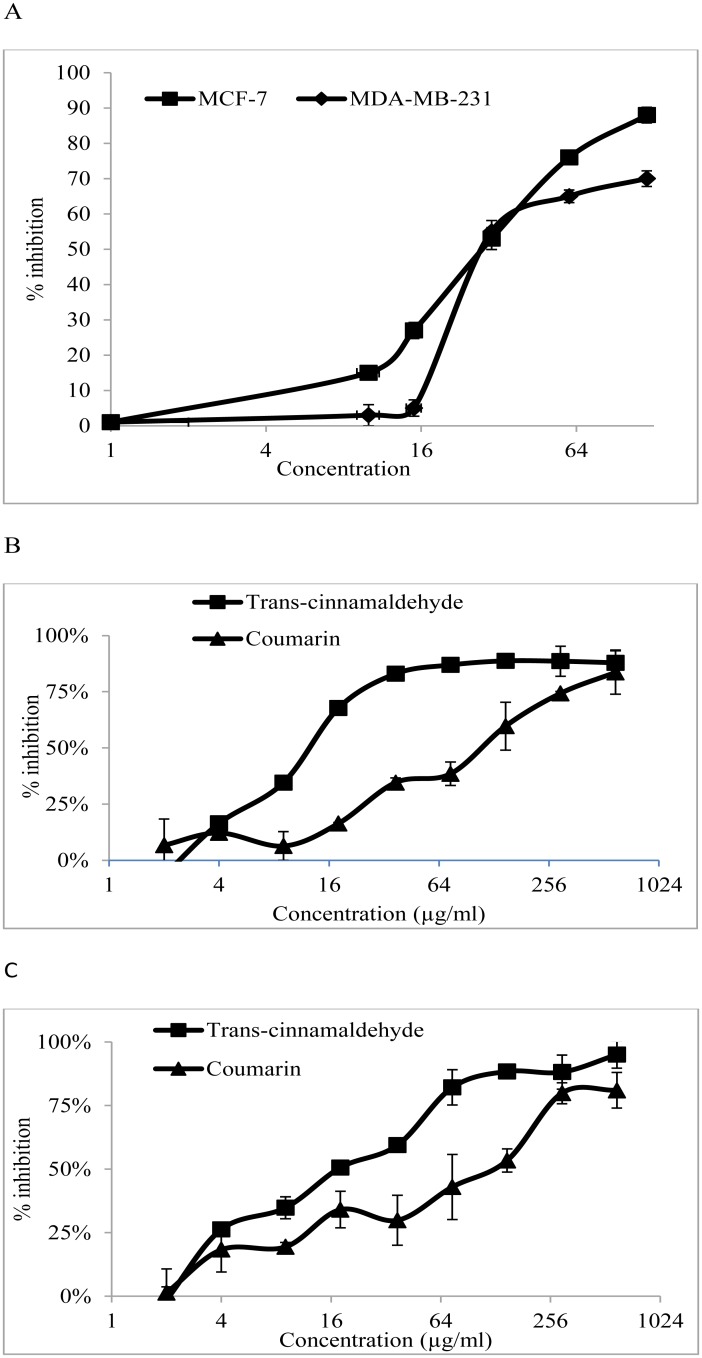
Antiproliferative activity of CE, *trans*-cinnamaldehyde and coumarin in MDA-MB-231 and MCF-7 cells. (A) The % inhibition when MDA-MB-231 and MCF-7 cells were exposed to various concentrations of the CE. (B) The % inhibition when MDA-MB-231 cells were exposed to various concentrations of *trans*-cinnamaldehyde and coumarin. (C) The % inhibition when MCF-7 cells were exposed to the various concentrations of *trans*-cinnamaldehyde and coumarin. Results are expressed as mean ± std. dev. (n = 3). IC_50_ is defined as the concentration of extract that inhibited 50% of cell proliferation. ND = not detected.

CE was applied at different doses on MCF-7 and MDA-MB-231 cells. Using the MTT assay, the anti-proliferative activity of the CE in the cell lines was obtained. The results (Fig 1A) show that CE had IC_50_ values of 34±3.52 and 32.42±1.37 μg/ml in MCF-7 and MDA-MB-231 cells, respectively, indicating that the two cell lines are almost equally sensitive to CE.

### CE disrupts the activity of antioxidant enzymes

**Fig 2:**
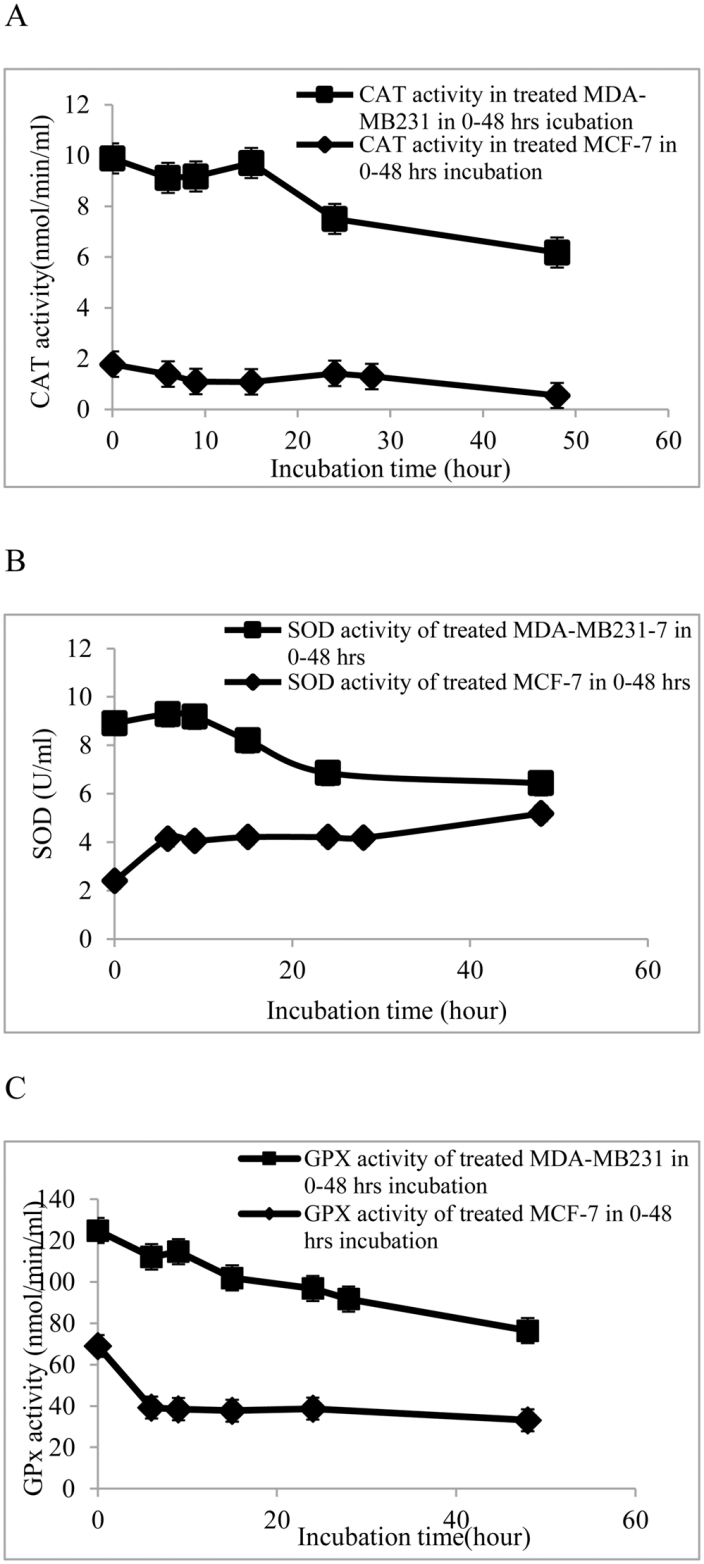
Activities of antioxidant enzymes in MDA-MB-23 and MCF-7 cells treated with 100 μg/ml of CE. Breast cancer cell lines, MCF-7 and MDA-MB-231, were seeded into 12-well plates containing RPMI 1640 and DMEM, respectively, and supplemented with 10% FBS at 5 × 10^6^ cells/well and allowed to attach for 24 h. The cells were treated with 100 μg/ml of CE (IC_70_ concentration determined from MTT assay) at varying time points (6, 9, 12, 24, and 48 h incubation). Activity of (A) catalase, (B) superoxide dismutase and (C) glutathione peroxidase was determined using commercial assay kits. Results are expressed as mean ± standard deviation. P < 0.05 compared to the control (without extract) as tested by the Student’s *t*-test.

Inducing oxidative stress to suppress cancer cell proliferation is a key mechanism in many chemotherapy drugs [22, 23]. Antioxidant enzymes like superoxide dismutase (SOD), catalase (CAT) and glutathione peroxidase (GPx) play key roles to maintain this balance. Any disruption in expression or activity of these enzymes would impose greater oxidative stress on the cell and metabolic reactions [23]. Therefore, to investigate the antiproliferative mechanism of CE the activity of these enzymes were measured in this study (Fig 2).

In Fig 2, it can be seen that the antioxidant enzymes reacted differently to CE treatment in MCF-7 and MDA-MB-231 cells. In MCF-7 cells the activity of SOD increased up to two times within the first 10 h, while GPx and CAT activity slightly decreased at the same time. Finally, SOD increased by 3.5 times in 48 h, while GPx and CAT decreased to 0.65 and 0.6 times of initial values, respectively.

In MDA-MB-231 cells in the first 10 h, SOD slightly increased while GPx and CAT dropped down to 0.57 and 0.5 times of their initial values. Subsequently, SOD decreased to 0.67 while GPx and CAT experienced faster reduction to 0.54 and 0.125 times of their initial values. This is the first time that a difference in the response of the antioxidant enzymes in both cell lines has been reported.

### CE disrupts the ROS generated in MCF-7 and MDA-MB-231 cancer cells

Intracellular ROS generation was evaluated using intracellular peroxide-dependent oxidation of DCFHDA to form fluorescent DCF. H_2_O_2_ was used as a positive control.

**Fig 3:**
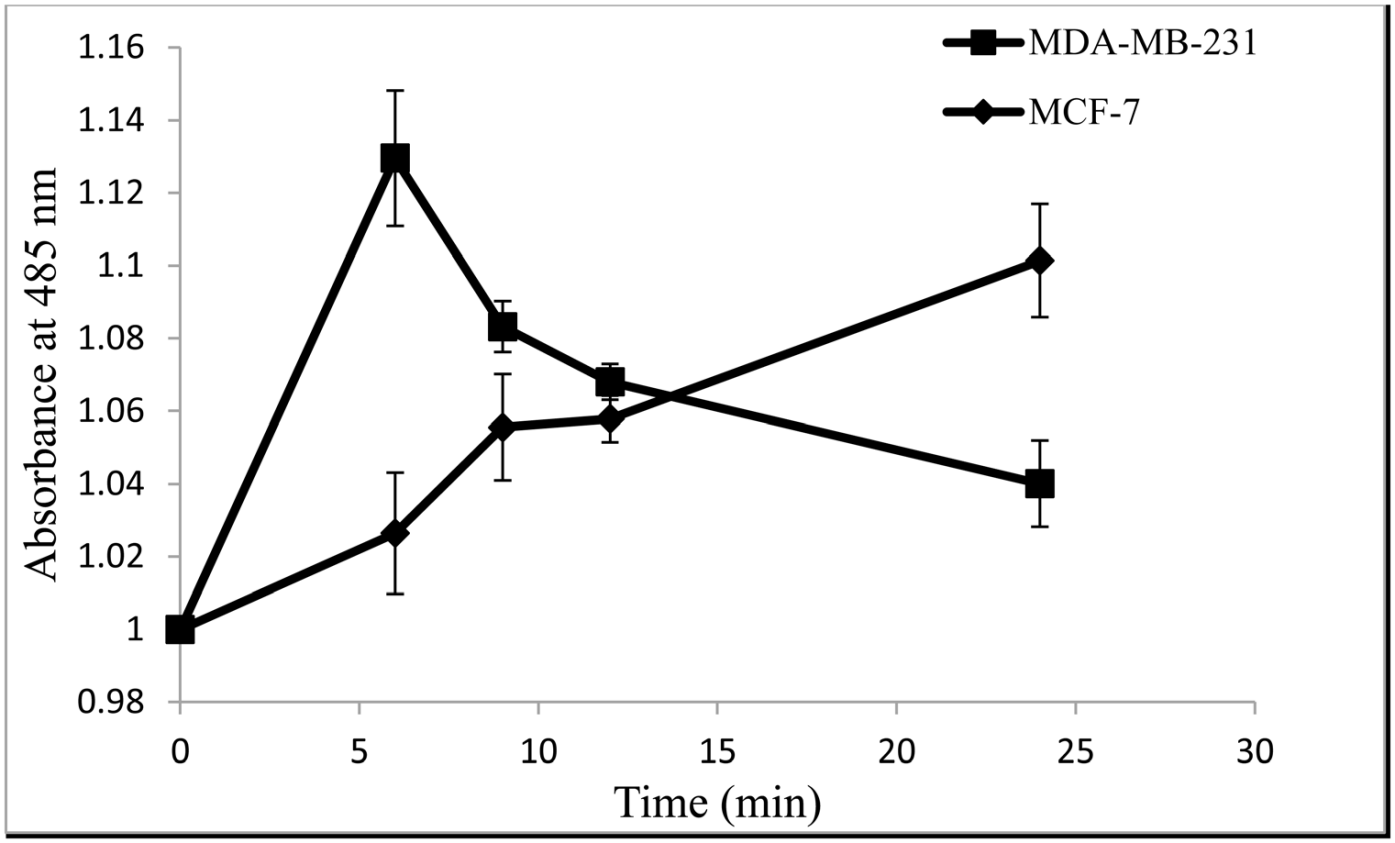
Intracellular ROS generated in MCF-7 cells and MDA-MB-231cells treated with CE at varying time points. Cells were treated with the IC_50_ concentration (35 μg/ml) of CE and incubated for 6, 9, 12, 24, and 48 h. Results are expressed as mean ± standard deviation. P < 0.05 compared to the control (without extract) as tested by the Student’s *t*-test.

Fig 3 shows that when MCF-7 cells were treated with CE, the intracellular ROS increased by 10.1% compared to the untreated cells. At 6 h, MCF-7 cells did not show a significant difference with the non-treated cells but the ROS level increased steadily afterward. In MDA-MB-231 cells, the ratio of ROS increased immediately after treatment and increased to 13% which then decreased to 4% at 24 h.

### CE induces apoptosis in MCF-7 and MDA-MB-231 cancer cells

Microscopic observation of the cells showed a rapid cell death in the treated cells by CE. Apoptosis and necrosis are two leading causes of cell death explaining the pronounced effect of anti-proliferative activity. Apoptosis can be differentiated from cytotoxicity in many ways such as DNA condensation or fragmentation, cell blebbing, changes in cell-membrane permeability [24] and filipping of phosphatidylserine to the extracellular (outer) surface of the cell [25]. Using fluorescence microscopy and the two dyes of AO/PI, and also Annexin V the occurrence of apoptosis or necrosis were qualitatively and quantitatively investigated. The extract was found to induce apoptosis in both cells, MCF-7 and MDA-MB231.

**Fig 4:**
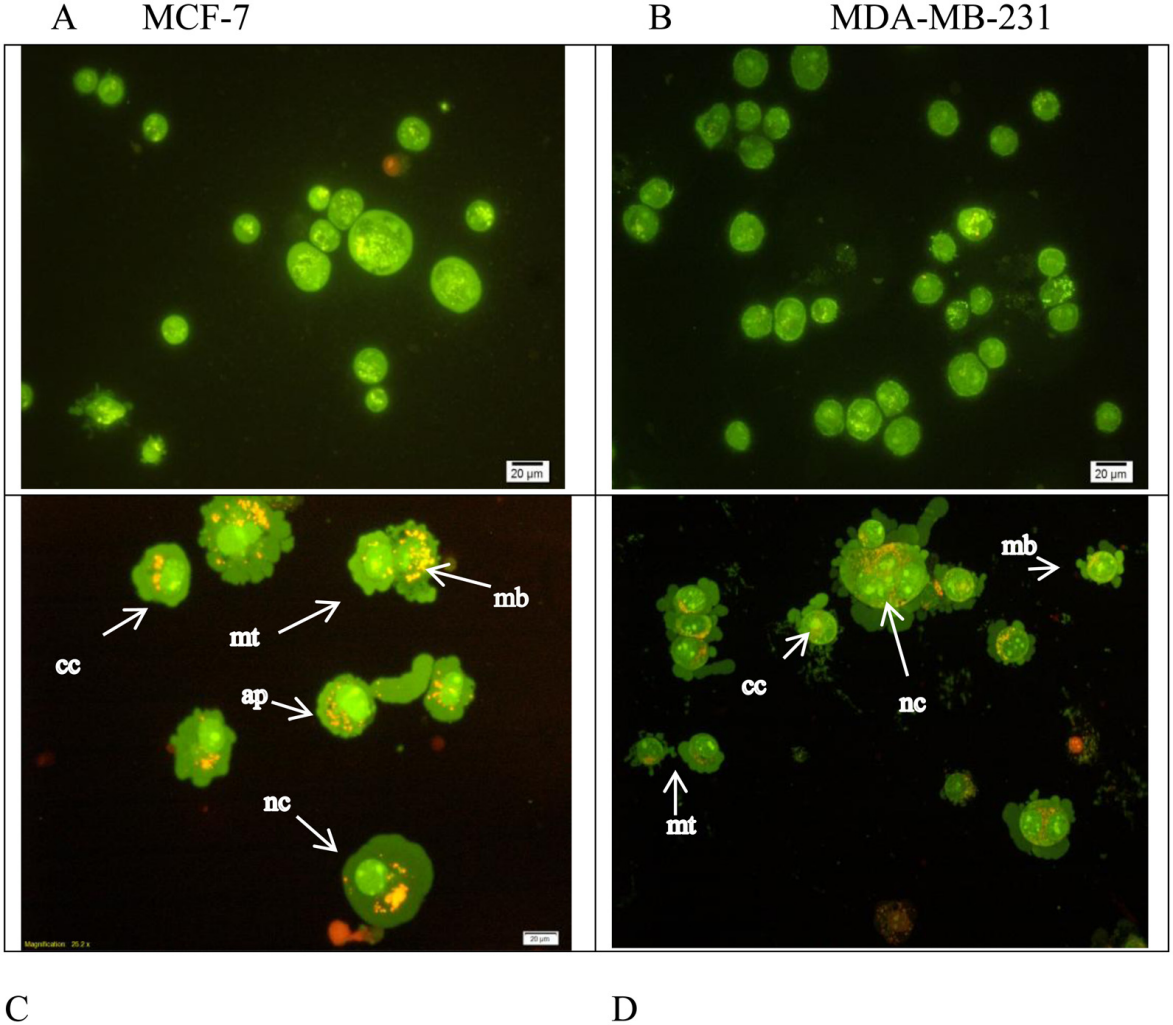
Morphological assessment of CE-treated and untreated MCF-7 and MDA-MB231 cells. A double fluorescent dye (AO/PI) staining method was used on cells after 24 h incubation with the IC_50_ concentration of CE. Apoptosis characteristics such as, early apoptosis (ap) nuclear compaction (nc), chromatin condensation (cc), the membrane plasma blebbing (mb) and the mitotic cells (mt) were observed in CE-treated (C) MCF-7 and (D) MDA-MB231 cells using an Olympus BX50 fluorescence microscope. Untreated controls (A) MCF-7 and (B) MDA-MB-231 were included. (200 X) magnification morphology.

After 24 h treatment with CE at the IC_50_ concentration, cells exhibited cytoplasmic protrusions. In most of the treated cells (either MCF-7 or MDA-MB-231 cells), cell membrane blebbing and dense green nuclei indicated nuclear chromatin condensation as a sign of apoptosis. This study indicated that apoptosis is the main cause for the anti-proliferative effect of CE on both cell lines (Fig 4).

**Fig 5:**
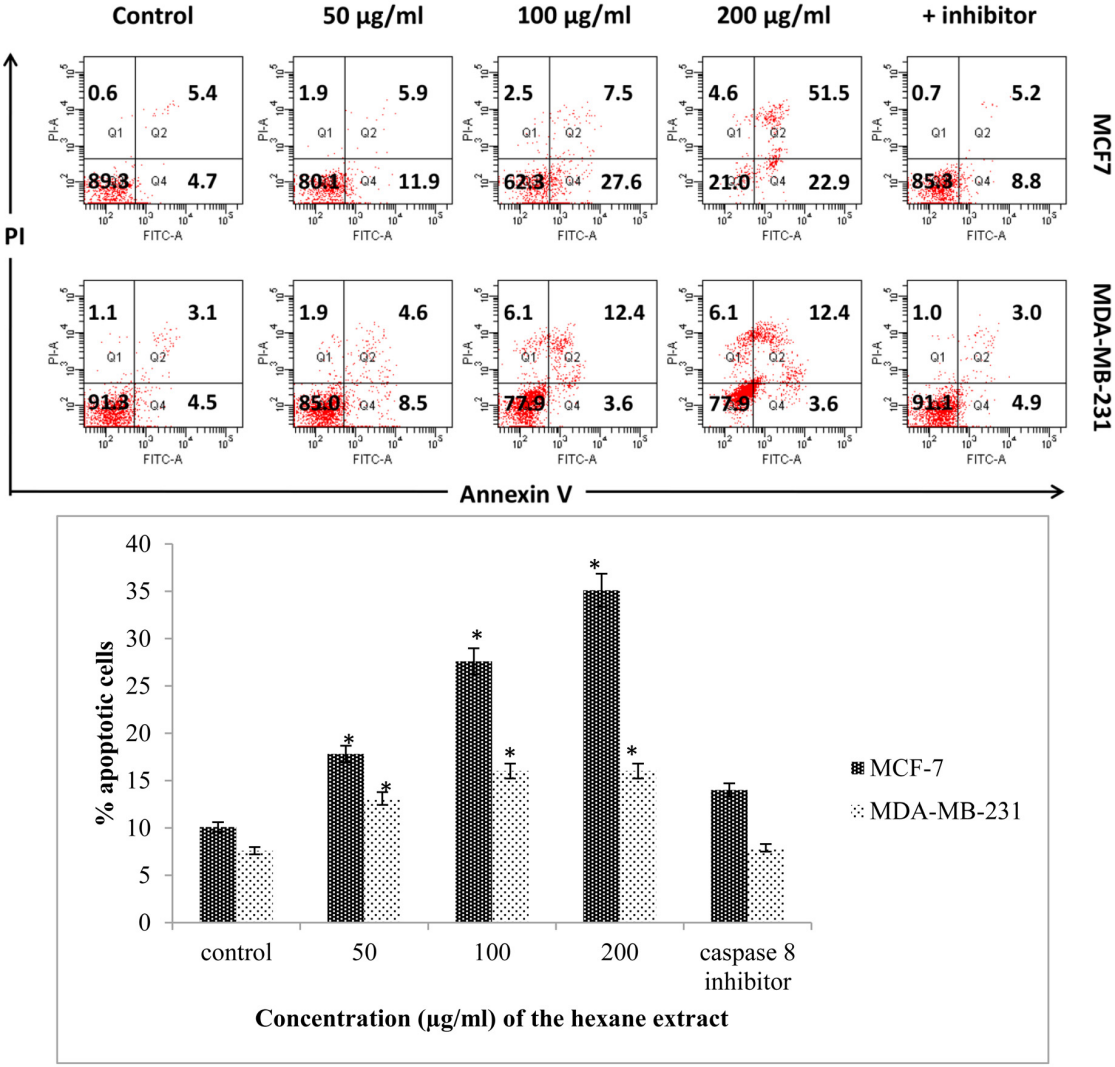
CE induces apoptosis in breast cancer cells. (A) MCF-7, MDA-MB-231 cells were stained with Annexin V/PI and subjected to flow cytometric analysis. The four quadrants represent living cells (Annexin V-PI-), early apoptotic (Annexin V+PI-), late apoptotic (Annexin+PI+) or necrotic (Annexin V-PI+) stages. (B) Percentage level of apoptotic cells when the cells were treated with different concentrations of CE. Values shown are percentages of each quadrant. *P<0.05, in comparison to control. The development of color was examined by bright field microscopy. (Magnification 200X). Z-VAD-FMK (caspase-8 inhibitor) was used as a negative control.

To confirm the occurrence of apoptosis quantitatively, the experiment was performed using apoptosis inhibitor on both treated MCF-7 and MDA-MB-231 cells stained with Annexin V/propidium iodide (PI) and then analyzed using flow cytometry. The results showed that Annexin V+/PI—apoptotic cell population increased in a dose-dependent manner (approximately 10% to 35%) in MCF-7 cells and approximately 8% to 15% in MDA-MB-231 cells treated with CE compared to the control (Fig 5).

Using the apoptosis inhibitor, Z-VAD-FMK, no significant changes in mortality were observed between the treated cells with both CE and the inhibitor with that of the control. This agrees with the induction of apoptosis through the caspase cascade in treated MCF-7 and MDA-MB-231 cells with CE.

### CE-induced apoptosis is mediated by caspase 8

**Fig 6:**
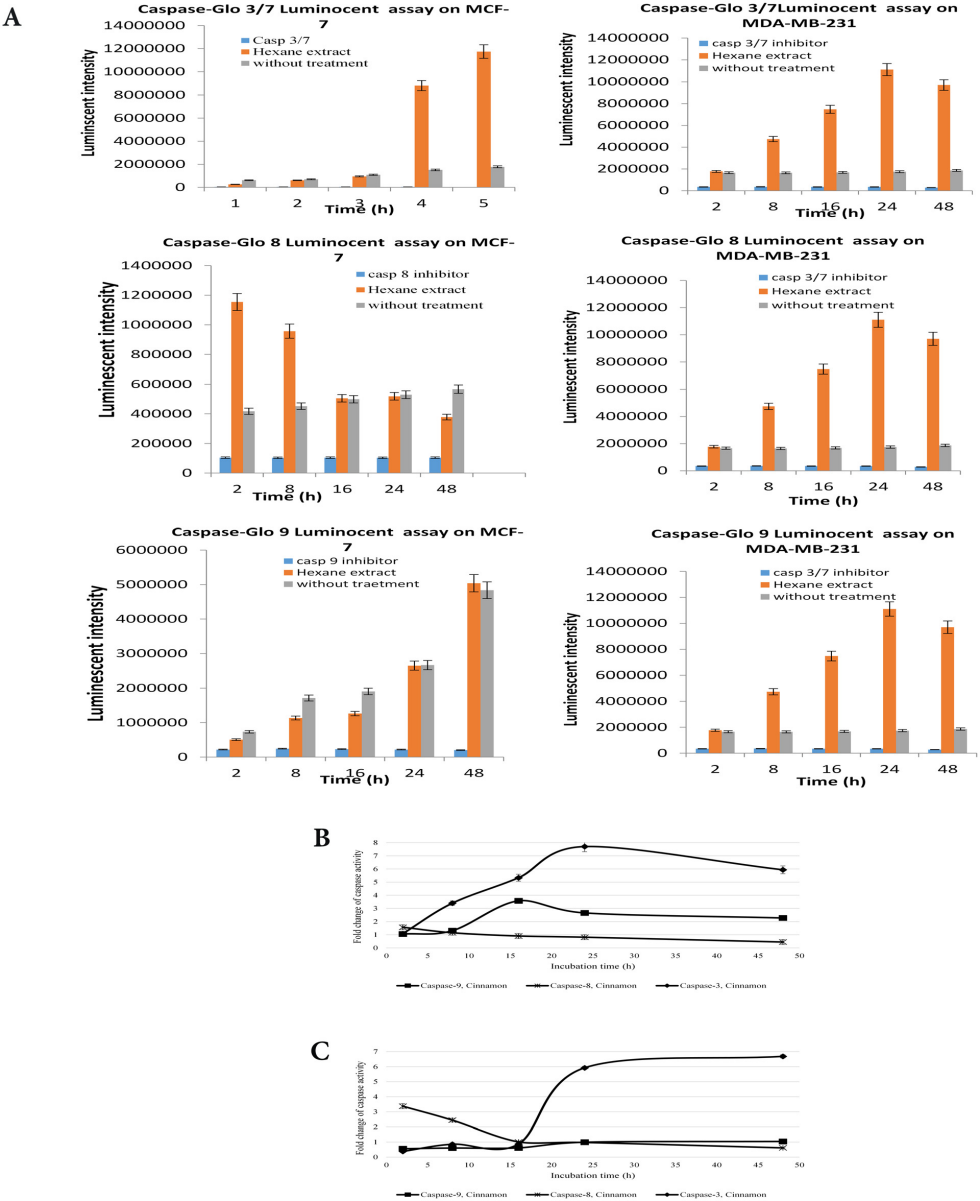
The Caspase-Glo luminoscence of MCF-7 and MDA-MB-231 cells treated with CE, without treatment, and treated with caspase inhibitor. Cells were treated with the IC_50_ concentration (35 μg/ml) of CE at varying time points (2, 8, 16, 24, 28 h). (A) The Caspase-Glo luminoscence of MCF-7 and MDA-MB-231cells treated with CE, without treatment and treated with caspase inhibitor. (B) The fold change in caspase activity of MCF-7 and (C) MDA-MB-231cells treated with CE. Results are expressed as mean ± standard deviation. P < 0.05 compared to the control (without extract) as tested by the Student’s *t*-test.

Caspases act in concert with a cascade triggered by apoptosis signaling [26]. There are two main apoptosis pathways namely the death receptor pathway which is mediated by caspase-8 and the mitochondrial pathway which is mediated by caspase-9 and both are followed by activation of the executioner caspases -3 and -7. Assessment of the activity of caspase-3/7, -8 and -9, indicated that CE activated caspase-8 in MCF-7 and MDA-MB-231 cells (Fig 6).

In both MDA-MB-231 and MCF-7 cells, caspase-8 activity increased almost immediately after the treatment with CE up to 1.57 and 3.38 folds at 2 h, respectively. Following the activation of caspase-8, caspase-3/7 were activated in both cell lines up to 7.70 and 5.92 folds in MDA-MB-231 and MCF-7 cells in 24 h, respectively.

It was noted that the activation of caspase-3/7 in MCF-7 cells was initiated with a considerable delay compared to MDA-MB-231 cells. Also, the measurement of caspase-3/7 activity in MCF-7 cells before 24 h indicated a sort of suppression compared to control cells.

Interestingly, caspase-9 did not exhibit the same pattern in MCF-7 and MDA-MB-231 cells. Caspase-9 was activated (3.58 folds at 16 h) only in MDA-MB-231 cells but not in MCF-7 cells. Caspase-9 seemed to be suppressed by the CE treatment. A similar pattern was observed with caspase-3/7 up to 16 h. In other words, the activity of caspase-9 in the treated cells is even less than occasional caspase-9 activity in untreated cells (Fig 6).

### CE alters apoptosis-related gene expression differently in MCF-7 and MDA-MB-231 cells

Apoptosis is a complicated but very well controlled pathway. More than a hundred genes are directly or indirectly involved in controlling and performing apoptosis. In this study the expression level of some of the more important genes in apoptosis were investigated using real-time RT-PCR. It revealed, for the first time, that the gene expression patterns was clearly different in MCF-7 cells compared to MDA-MB-231 cells treated with CE.

**Fig 7:**
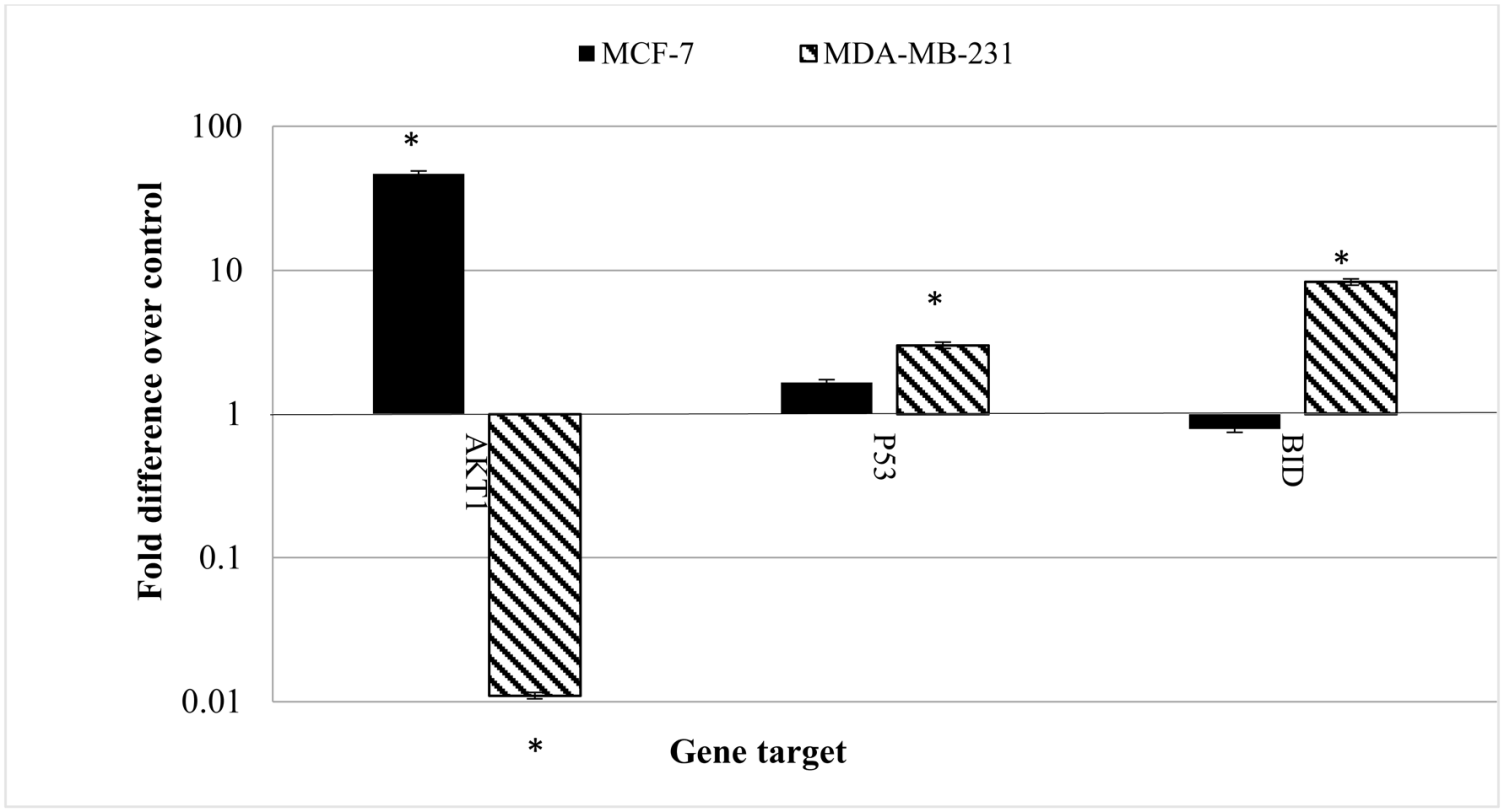
The effect of CE on gene expression in MCF-7 and MDA-MB-231 cells. Both cell lines were treated with the IC_50_ concentration (35 μg/ml) of CE for 24 h. Total RNA was extracted and cDNA was synthesized using commercial kits. Real-time RT-PCR was performed and the relative expression of the genes was calculated using the delta-delta Ct method and normalized with 18S eukaryotic rRNA. Results are expressed as fold variation over carrier control (blank). Results are expressed as mean ± standard deviation. Statistical significance was calculated based on the mean ΔCt values by the Student’s *t* test. *Indicates significant differences from untreated cells (p < 0.05).

Fig 7 shows the fold change of the gene expression in MCF-7 and MDA-MB-231 cells treated with CE. The most distinctive pattern was measured in AKT1 expression. AKT1 in MCF-7 cells was significantly over-expressed (46.617 fold) while it was down-regulated in MDA-MB-231cells (0.011 fold). The expression of p53 and BID were also different in the two cell lines. While the expressions of the named genes were not significantly altered in MCF-7 cells, they were over-expressed (2.7 and 8 folds) in MDA-MB-231 cells, respectively.

### Effects of CE on AKT1 protein expression in MCF-7 and MDA-MB-231cells

**Fig 8:**
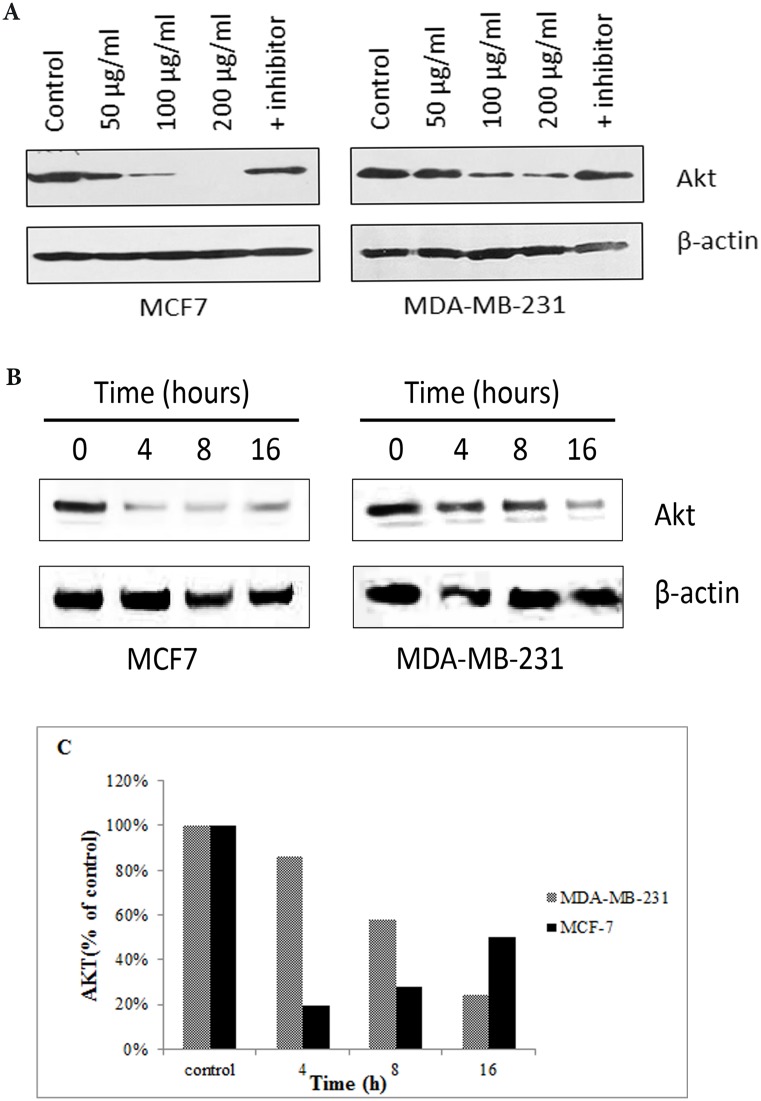
The effect of CE on AKT1 protein expression in MCF-7 and MDA-MB-231 cells using Western blot analysis. (A) Western blot analysis of the expression level of AKT1 in MDA-MB-231 and MCF-7 cells treated with different concentrations of CE, and caspase-8 inhibitor. AKT1 is down-regulated in all the treatments, except with the caspase-8 inhibitor. (B) Western blot analysis of the expression level of AKT1 in MDA-MB-231 and MCF-7 cells treated with the IC_50_ (35 μg/ml) of CE with time was carried. It was demonstrated that in MDA-MB-231, the expression of AKT1 was reduced in a time-dependent manner. (C) In MCF-7 cells, the expression level of AKT1 decreased dramatically from 0–4 h and then gradually increased by 31% from 4–16 h. In MDA-MB-231 the expression level of AKT1 decreased from control to 16 h by 76%. The blots were scanned and analyzed using ImageJ software.

Fig 8A shows that the protein expression of AKT1 decreased in both cell lines in a concentration dependent manner. But no change was observed in the expression level of AKT1 between control and the cells treated with the CE+inhibitor. In MDA-MB-231 cells, the expression of AKT1 was found to be reduced in a time-dependent manner, while MCF-7 cells decreased dramatically from 0–4 h and then steady increased from 19% at 4 h to 48% at 16 h (Fig 8B).

### Isolation and identification of coumarin and *trans*-cinnamaldehyde in CE

**Fig 9:**
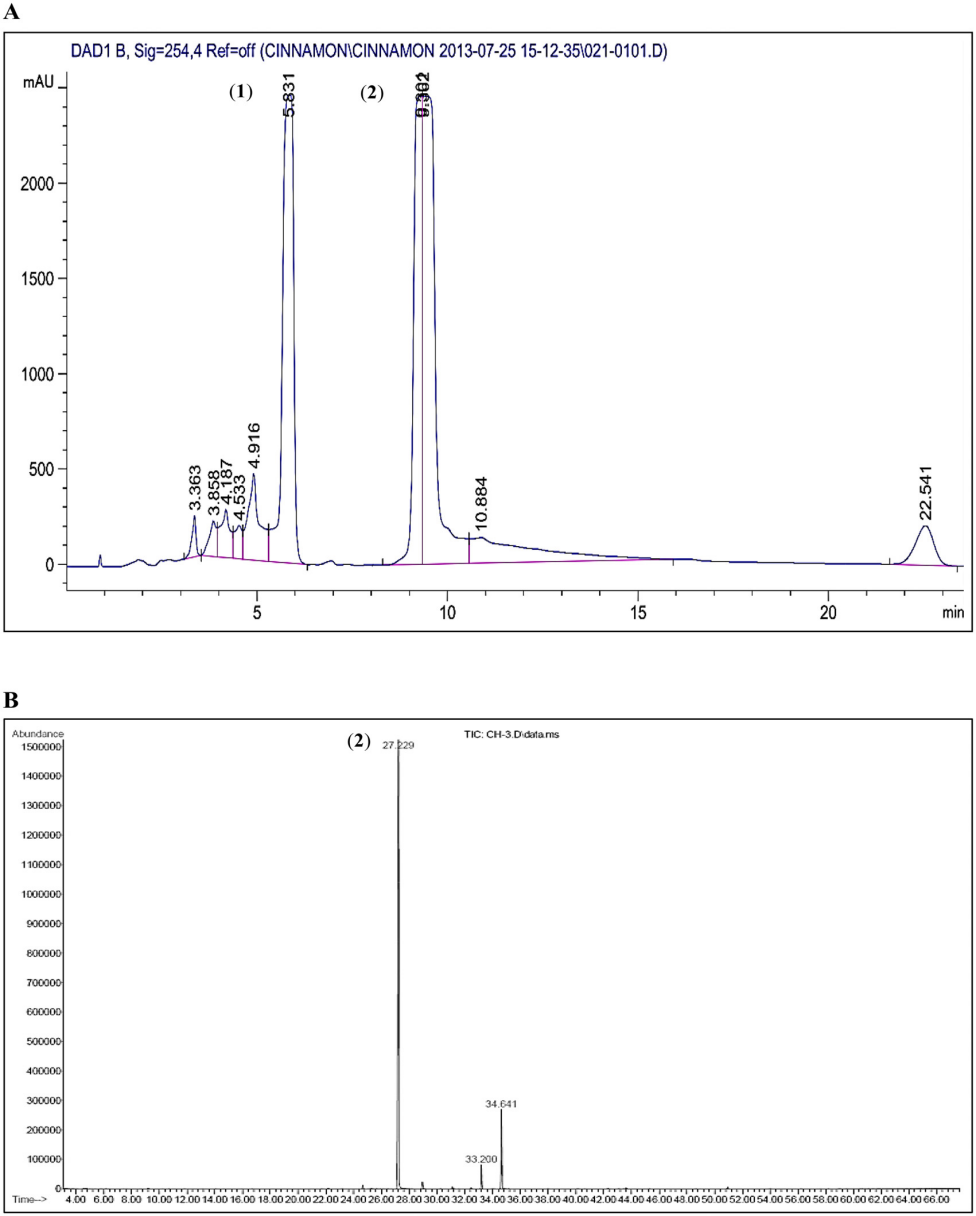
Semi-preparative HPLC chromatogram of CE and GCMS total ion chromatogram profile. (A) The peaks were detected at UV wavelength 254 nm. The two isolated peaks were identified as coumarin (1) and *trans*-cinnamaldehyde (2) through mass spectral library. (B) The two major peaks were identified as coumarin (1) and *trans*-cinnamaldehyde (2) (Wiley 9^th^ edition NIST11 Mass Spectral Library, USA).

Semi-preparative HPLC of CE was performed and the major peaks were isolated. The isolated compounds, identified using GC-MS, were coumarin and *trans-*cinnamaldehyde according to the W9N11 library of database (Fig 9).

The anti-proliferative activities of both the identified compounds were assayed using the cell proliferation assay. *Trans*-cinnamaldehyde exhibited a strong anti-proliferative activity towards MCF-7 and MDA-MB-231 cells with IC_50_ values of 9.61±0.07 and 14.21±0.02 μg/ml, respectively. Coumarin cytotoxicity was weaker than *trans*-cinnamaldehyde with IC_50_ values of 107.98±0.05 μg/ml in MDA-MB-231 cells and 98.15±0.04 μg/ml in MCF-7 cells (Fig 1B & 1C).

## Discussion

*Cinnamomum cassia* extract (CE) has been investigated for its apoptotic effect on a variety of cell lines. However, the molecular mechanism of the observed apoptosis is not well described. In the present study, CE inhibited the proliferation of the breast cancer cell lines, MCF-7 and MDA-MB-231.

Morphological observation showed occurrence of apoptosis in both cell lines which was further supported by fluorescence microscopy and flow cytometry. Cell blebbing, DNA condensation and fragmentation, followed by changes in cell membrane permeability to PI dye were observed in both cell lines (Figs 4 and 5). Increasing activity of caspase enzymes, including caspase-3/7, -8 and -9 (Fig 6) further proves the induction of apoptosis upon CE treatment.

Flow cytometric annexin V analysis of CE treated cells in the presence and absence of caspase inhibitor further elaborates the role of caspase cascade apoptosis in the antiproliferative effect of CE [27].

SOD scavenges superoxide anion and produces hydrogen peroxide which is subsequently detoxified to water by GPx or CAT [28]. A relative decrease in the GPx and CAT ratio to SOD activity would result in oxidative stress due to hydrogen peroxide accumulation in the cell [23]. It has been reported that a high level of H_2_O_2_ produced by increasing SOD activity or decreasing GPx and CAT activity leads to H_2_O_2_ accumulation and cancer cell death [29, 30]. Also, it has been reported that quinoline derivatives inhibit the growth of MCF-7 cells by increasing the activity of superoxide dismutase and suppressing CAT and GPx activities [31].

In our study, on treatment of the breast cancer cell lines with CE, the SOD ratio to CAT and GPx was increased in both cell lines, leading to accumulation of hydrogen peroxide. Similar results are reported for other plants extracts, such as *Coriandrum sativum* and other phenolic compounds [32–34]. Though the exact mechanism of disruption of antioxidant enzymes by CE is not clear, the results indicate evidence of oxidative stress in the breast cancer cell lines following the treatment. In 2003, Huang et. al. showed that the oxidative stress would initiate apoptosis [35].

When MCF-7 and MDA-MB-231 cells were treated with CE, the intracellular ROS increased by 10.1% and 13%, respectively, but in different timing patterns. The ROS level almost immediately increased in MDA-MB-231 cells after treatment whereas it steadily increased in MCF-7 cells after 6 h. The difference in the ROS ratio supports the idea that the two cell lines were responding to the treatment with different mechanism. ROS especially increased when the cells were going through apoptosis mediated with mitochondrial membrane disruption (caspase-8 mediated apoptosis) [36].

There are two main apoptosis pathways, namely the death receptor pathway which is mediated by caspase-8, and the mitochondrial pathway which is mediated by caspase-9. Both caspase-8 and -9 activate the executioner caspase-3/7. The observed chronological pattern of the activation of the caspase enzymes in this study suggests that the death receptor pathway predominantly mediates apoptosis in both cell lines. In the death receptor mediated apoptosis, FADD-caspase-8 pathway, caspase-8 activation would either activate caspase-3/7 directly or through caspase-9 or simultaneously [37]. The exact mechanism of simultaneous activation of both pathways has yet to be elucidated but is reported frequently in cancer drug discovery research [35, 38, 39].

In this study in particular, caspase-8 was activated immediately after the treatment and followed by caspase- 3/7 activation. Caspase-9 was only activated in MDA-MB-231 and not in MCF-7 cells. Although the absence of caspase-3 in MCF-7 might trigger the caspase-9 activity as suggested by Blanc et. al., the lower activity of caspase-9 in the treated cells compared to the untreated cells suggests an active suppression of caspase-9 in MCF-7 cells (Fig 6) [40]. It is interesting to note that caspase-3/7 and -9 were suppressed in the treated MCF-7 cells up to 16 h. This corresponds to the data obtained from gene expression studies (discussed below). The almost similar sensitivity of both cell lines to CE treatment despite the activation of caspase-9 in MDA-MB-231 suggests that the caspases-3/7 activation by caspase-8 plays the main role in apoptosis and overshadows caspase-9 activation. Although caspase-8 activation is mostly known as the extrinsic apoptosis pathway it has been shown that oxidative stress could initiate apoptosis mediated by activating a FasL-independent FADD-caspase-8 pathway [35].

The two cell lines also responded to the CE treatment differently at gene expression level. The gene expression study of some of the main genes involved in apoptosis showed the over-expression of AKT1 in MCF-7 cells. AKT1 acted as an apoptosis inhibitor through phosphorylation of pro-caspase-9. On the other hand, AKT1 was down-regulated in MDA-MB-231 cells which made the cells more susceptible to apoptosis inducers. P53 and BID were over expressed in MDA-MB-231 cells which were involved in the activation of caspase-9 through mitochondrial disruption [41, 42].

The p53 gene encodes the tumor protein p53, a transcriptional factor that binds to DNA and activates the expression of downstream genes that inhibit growth of cells and enforces apoptosis [43]. The data from this study suggests that in MDA-MB-231 cells, the down regulation of AKT1 would increases the cell susceptibility to apoptosis. The up regulation of p53 increased by DNA oxidative damage would initiate apoptosis through the mitochondrial pathway [44].

The pattern of the activity of caspases together with the observed changes in gene expression can be interpreted as follows: BID is a member of the Bcl2 family, and a specific proximal substrate of caspase-8. Upon cleavage by caspase-8, BID translocates to the mitochondria and releases cytochrome c in the cytosol which itself initiates apoptosis through activation of apoptosomes and caspase-9 [45]. AKT1 is a gene which improves cell survivability in many ways including blocking caspase-9 by phosphorylation of pro-caspase-9 [46]. BID mediates the activation of caspase-9 by caspase-8 through the mitochondrial pathway. Therefore, the over expression of BID concurs with the pathway of caspase-9 activation by caspase-8 [9].

Treating the cells with different concentrations of CE showed that, despite the results obtained from gene expression studies, AKT1 was drastically down regulated unless the cells were treated with apoptosis inhibitor. It was shown that down-regulation of AKT1 at protein level upon CE treatment was a crucial event in apoptosis which is supported by the anti-proliferative effect of CE in both cell lines.

On the other hand, treating cell lines at IC_50_ concentrations at different times (0–16 h) revealed that the AKT1 was over-expressed from 4 to 16 h after a drastic down-regulation at 4 h in MCF-7 but not in MDA-MB-231 cells. Although this steady increase of AKT1 protein expression from 4 h to 16 h (recovering up to 46% of AKT1 expression) is lower than the huge drop at 4 h, it confirms that the observed over-expression at gene expression level has some effects on the protein level for MCF-7 but not in MDA-MB-231 cells.

The gene and protein expression data of AKT1, after concentration- or time-dependent CE treatment, suggests the double effect of CE on MCF-7 cells but not in MDA-MB-231. This double effect is probably through more than one particular pathway. The dominant and fatal effect of CE through which apoptosis occurs was pronounced and confirmed in this study in different ways, but the cell-stabilizing effect of CE, up-regulation of AKT1 from 19% to 46% in 4–16 h after treatment, is being reported for the first time.

The obvious difference in AKT1 gene and protein expression response to CE in MCF-7 and MDA-MB-231 cells could be a key reason to the differences previously observed in caspase enzyme activity. While caspase-9 was activated in MDA-MB-231 cells by more than 3 folds, the caspase-9 activity in MCF-7 cells was less than that of the untreated cells (0.55 folds). The late activation of caspase-3/7 after the activation of caspase-8 can be explained by the inhibitory effect of AKT1 on cell death. Some studies have shown that AKT1 suppresses p53, BID and caspase-9 [47–49]. It has to be noted that the proposed pathways are yet to be confirmed by direct assessment.

Despite the high level of AKT1 expression and its consequent effect on caspase-9 suppression, MCF-7 cells do not show a considerable difference in mortality in response to CE. This can be explained by the mechanism of FasL-independent FADD-caspase-8 apoptosis activation. It is shown that the accumulation of hydrogen peroxide induces apoptosis through caspase-8 [35]. Caspase-8, upon activation, would directly initiate caspase-3/7 as the executioner apoptosis enzymes. Also, caspase-8 would activate caspase-9 through the mitochondrial pathway [50] which finally leads to the activation of caspase-3/7. In MCF-7 cells, the over expression of AKT1 blocked the mitochondrial pathway but was not significantly effective on the mainstream pathway, based on the differences between MCF-7 and MDA-MB-231 cells but the exact mechanism is not clear in this study. MCF-7 cells are estrogen-responsive while MDA-MB-231cells are estrogen-receptor negative, which means that over-expression of estrogen receptors on the cell membrane of MCF-7 cells would respond to a very small amount of estrogen (or estrogen-like compounds) and trigger various cellular pathways. One possible explanation to the over expression of AKT1 could be the presence of an estrogen-like compound in CE. This idea is supported by other reports [51–53]. It should be stressed that the differences between MCF-7 and MDA-MB-231 cells are not limited to their response to estrogen alone, but are distinguishable with a couple of other characteristics as well, such as response to progesterone, mutation at HER2 and others, which calls for more research in this area.

The active compounds in CE were isolated, identified as coumarin and *trans*-cinnamaldehyde and validated for their bioactivity. The apoptotic effect of *trans*-cinnamaldehyde has been studied on several cancers including colon, breast [43], blood [54, 55], lung, skin, kidney, ovarian and prostate [56]. Coumarin is also known as an apoptosis inducer compound in several cancers like cervical [57], lung [58], gastric [59] and blood [60].

No proliferative compound was identified or isolated from CE. But the difference in sensitivity to CE in MCF-7 and MDA-MB-231 cells compared with *trans-*cinnamaldehyde (the main apoptotic compound) suggests the possible elimination of the anti-apoptotic compound of CE during the purification procedure. It is suggested that these compounds might either be present at very low concentrations and not detectable by the methods used in this study or were degraded in the purification procedure.

These findings greatly contribute to the understanding of the anti-tumor activity of CE in the two breast cancer cells (MCF-7 cells as an ER^+^ and MDA-MB-231 cells as an ER^-^).

**Fig 10:**
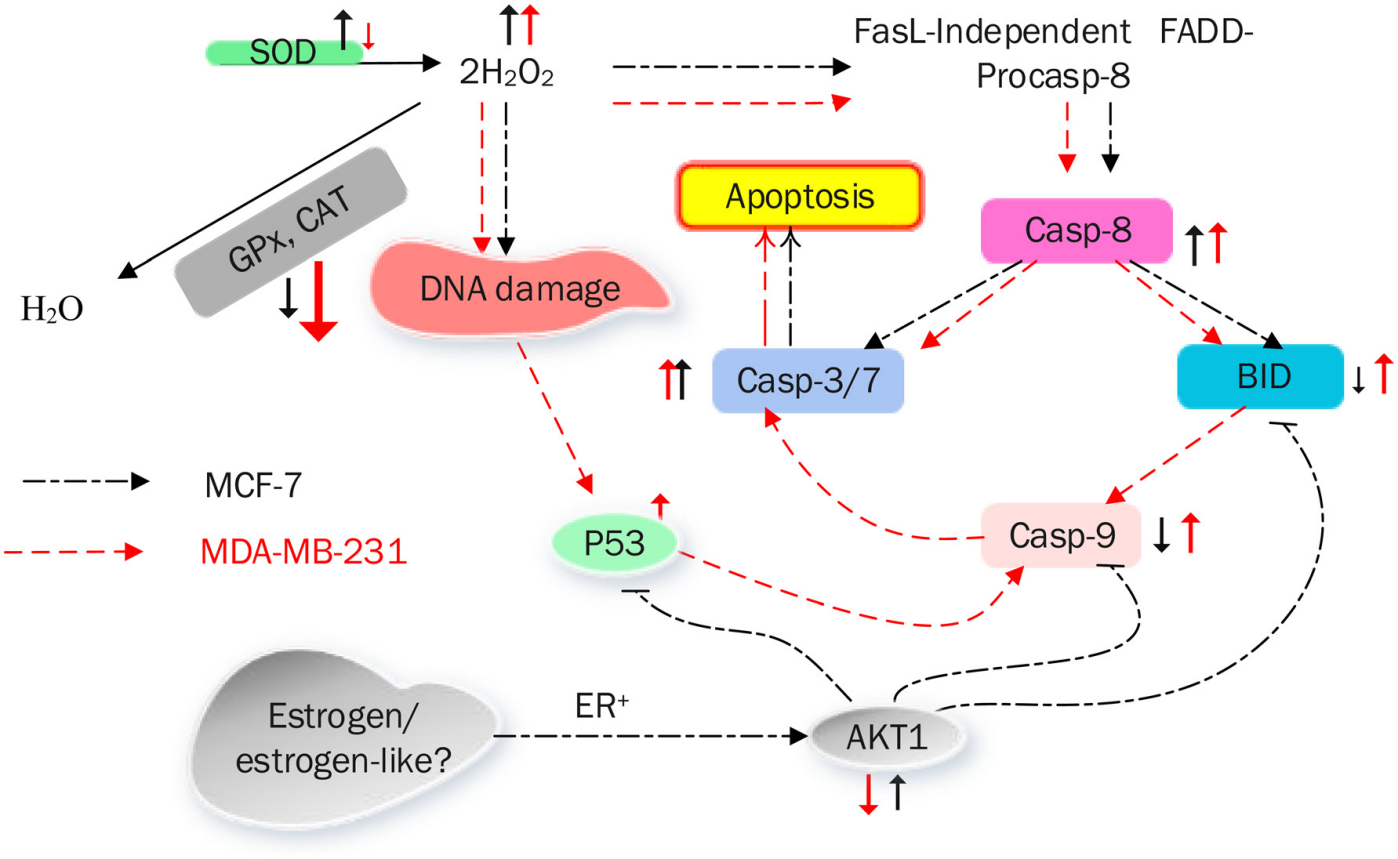
A model summarizing the mechanism of CE-induced apoptotic cell death in MCF-7 and MDA-MB-231 breast cancer cells.

Fig 10 summarizes the mechanism of CE-induced apoptotic cell death in MCF-7 and MDA-MB-231 cells.
